# *Bacillus subtilis* and surfactin inhibit the transmissible gastroenteritis virus from entering the intestinal epithelial cells

**DOI:** 10.1042/BSR20170082

**Published:** 2017-04-10

**Authors:** Xiaoqing Wang, Weiwei Hu, Liqi Zhu, Qian Yang

**Affiliations:** College of Veterinary Medicine, Nanjing Agricultural University, Weigang 1, Nanjing, Jiangsu 210095, People’s Republic of China

**Keywords:** antiviral, Bacillus subtilis OKB105, entry, surfactin, TGEV

## Abstract

Intestinal epithelial cells are the targets for transmissible gastroenteritis (TGE) virus (TGEV) infection. It is urgent to develop a novel candidate against TGEV entry. *Bacillus subtilis* is a probiotic with excellent anti-microorganism properties and one of its secretions, surfactin, has been regarded as a versatile weapon for most plant pathogens, especially for the enveloped virus. We demonstrate for the first time that *B. subtilis* OKB105 and its surfactin can effectively inhibit one animal coronavirus, TGEV, entering the intestinal porcine epithelial cell line (IPEC-J2). Then, several different experiments were performed to seek the might mechanisms. The plaque assays showed that surfactant could reduce the plaque generation of TGEV in a dose-dependent manner. Meanwhile, after incubation with TGEV for 1.5 h, *B. subtilis* could attach TGEV particles to their surface so that the number of virus to bind to the host cells was declined. Furthermore, our data showed that the inhibition of *B. subtilis* was closely related to the competition with TGEV for the viral entry receptors, including epidermal growth factor receptor (EGFR) and aminopeptidase N (APN) protein. In addition, Western blotting and apoptosis analysis indicated that *B. subtilis* could enhance the resistance of IPEC-J2 cells by up-regulating the expression of toll-like receptor (TLR)-6 and reducing the percentage of apoptotic cells. Taken together, our results suggest that *B. subtilis* OKB105 and its surfactin can antagonize TGEV entry *in vitro* and may serve as promising new candidates for TGEV prevention.

## Introduction

Transmissible gastroenteritis (TGE) virus (TGEV) is an enveloped virus that belongs to the coronaviridae family within the coronavirus genus [1,2]. It is the causative agent of porcine TGE, leading to vomiting, acute diarrhoea, dehydration and a nearly 100% mortality in suckling piglets [3]. Current vaccines, neither inactivated nor attenuated, cannot provide full protection to pigs [4]. Therefore, it is urgent to discover and develop a novel anti-TGEV candidate to reduce the economic losses caused by TGE.

Surfactin is a cyclic lipopeptide antibiotic and biosurfactant synthesized by *Bacillus subtilis* [5]. It consists of an anionic seven-membered peptide cyclo and a mixture of several hydrophobic β-hydroxy fatty acids with chain lengths of 13–15 carbon atoms [6]. By this amphiphilic structure, surfactin is one of the strongest biosurfactants. Studies on surfactin are focused on properties against phytopathogenic microorganisms, such as antibacterial [7], antifungal [8], inhibition of fibre clot formation [9] and antiviral [10,11] ability. Some reports showed that surfactin could inactivate various enveloped virus, like vesicular stomatitis virus (VSV, rhabdoviridae) and suid herpes virus type 1 (SHV-1, pseudorabies virus) , by inserting into the outer layer of lipid membrane bilayer so that the envelope disintegrates [12]. But whether surfactin has the activity against TGEV, an animal enveloped virus from coronaviridae family, remains poorly understood. Moreover, our colleagues had reported that *B. subtilis* could antagonize enteropathogenic *Escherichia coli* (ETEC) infection [13]. In the present study, we investigated the antiviral effects of *B. subtilis* OKB105 and its surfactin against TGEV entry in the intestinal porcine epithelial cell line (IPEC-J2) cells.

To explore the possible mechanisms, the effects of *B. subtilis* OKB105 and surfactin on viral infectivity as well as the impact on the receptors of TGEV, epidermal growth factor receptor (EGFR) and aminopeptidase N (APN), were investigated. Additionally, the toll-like receptors (TLRs) and the apoptosis of IPEC-J2 cells were also detected. Our results reveal that both the *B. subtilis* OKB105 and surfactin exhibit the suppressive activity against TGEV entry and may possibly serve as potential candidates to reduce the economic loss caused by TGE.

## Materials and methods

### Cells and virus

The IPEC-J2 cell lines (Guangzhou Jennio Biotech Co, Ltd., China) were maintained in Dulbecco’s modified Eagle’s medium nutrient (DMEM from Life Technologies, Shanghai, China) supplemented with 10% FBS (Gibco), 1% penicillin/streptomycin (Life Technologies) and 16 mM Hepes (Life Technologies) in a 37°C, 5% CO_2_ incubator. The TGEV strain SHXB (10^8^ plaque forming units (pfu) per ml (pfu/ml)) was kindly provided by the Jiangsu Academy of Agricultural Sciences. All infections were performed at a multiplicity of infection (MOI) of 0.01.

### Bacteria and surfactin

### Inhibition of *B. subtilis* or surfactin

*B. subtilis* 168 and OKB105 (donated by Prof Xuewen Gao from College of Plant Protection in Nanjing Agricultural University) were cultivated in Luria broth (LB), then after centrifugation, the bacteria were washed three times to remove excess LB. Finally, the viable *B. subtilis* were resuspended in DMEM to the designed concentration from 1.00E + 07 to 1.00E + 10 colony forming units (cfu) per ml (cfu/ml). *B. subtilis* OKB105 was a surfactin producer transformed from *B. subtilis* 168 [14,15].

Surfactin used in the present study was extracted from *B. subtilis* OKB105 according to the procedures of Xue-wen et al. [16]. The concentration of surfactin is over 95% detected by HPLC.

### Cellular toxicity assessment

Toxic effects of the *B. subtilis* and surfactin on IPEC-J2 cells were determined using the MTT viability assay [17]. Suspensions of 100 μl containing different amounts of *B. subtilis* ranging from 1.00E + 06 to 1.00E + 09 cfu/ml and concentrations of surfactin ranging from 2.00E – 06 to 2.00E – 01 mg/ml were added to IPEC-J2 cell monolayers in a 96-well plate (Corning Costar) for 2 h before washing away. Then 20 μl of MTT (1 mg/ml, Sigma) was added to the cells per well and incubated for 4 h at 37°C, the reaction was stopped by adding an equal volume of lysis buffer (50% DMSO and 20% SDS, pH 7.4). The absorbance was read at 570 nm. The cell survival rate was determined as the stimulatory index (SI) calculated according to the following equation: SI = (OD_infected well_ − OD_bank control_)/(OD_negative well_ − OD_bank well_). Mock-treated cells served as control. Each experiment was performed in triplicate.

Three setups focused on the suppressive effect against TGEV entry varying the treatment period. Briefly, monolayers of IPEC-J2 cells were treated with *B. subtilis* 168, OKB105 and surfactin for 1.5 h respectively, which was washed away before infection with TGEV for 1.5 h (pre-treatment assay), TGEV was added to the cell layer together with *B. subtilis* 168, OKB105 and surfactin respectively, during the 1.5 h infection period (co-treatment assay), virus was mixed with *B. subtilis* 168, OKB105 and surfactin respectively and incubated for 1.5 h at 37°C, aliquots were removed and diluted 1:10 with DMEM supplemented with 5% FBS to stop the effect of the surfactin and then sterile filtered through a 0.22 μm filter. Then the filtrate were added to the cell layer and incubated for 1.5 h (out-treatment assay). For the indicated time points, cells were washed three times and kept in medium containing 1% penicillin/streptomycin for 0.5 h to kill any viable bacteria that were left. After incubation, cells were washed three times, then re-suspended in TRIzol (Sigma) and stored at –80°C until analyses. As for the Western blotting, nuclear and cytoplasmic proteins were extracted and isolated using the Nuclear and Cytoplasmic Protein Extraction Kit (Beyotime, Jiangsu, China) [18] .

### Plaque assays

To assess the direct effects of *B. subtilis* or surfactin on TGEV, we performed a plaque formation assay [19]. Briefly, the virus was mixed with different concentrations of *B. subtilis* or surfactin and incubated for 1.5 h at 37°C, after exorcizing the surfactin effect and probiotics as described above. Two hundred fifty microlitres of filtrate were added to confluent monolayers of ST cells (the susceptible cell) grown in six-well tissue culture plates (1–2 × 10^6^ per well) and incubated for 1.5 h at 37°C. After washing, the cells were overlaid with 1640 medium containing 0.7% Sea-Plague agarose, 2% FBS and 1% penicillin/streptomycin. The plates were incubated at 4°C for 30 min to solidify the overlay medium. Cells were then grown at 37°C and 5% CO_2_ to allow plaque formation. Viral plaques were visualized by staining with 0.8% (w/v) Crystal Violet dye after 2-day incubation. Virus titres were calculated according to the following formula: Titre (pfu/ml) = number of plaques/volume of diluted virus added to the well × dilution factor of the virus used to infect the well in which plaques were enumerated. Virus without any treatment served as control and ST cells without addition of TGEV served as mock.

### Binding effects of *B. subtilis*

In order to examine the possible direct binding of virus by *B. subtilis*, we mixed *B. subtilis* 168 or OKB105 with TGEV (1.00E + 07 cfu/ml bacterial cells with different MOIs: 0.01, 0.1, 0.2) for 1.5 h. After centrifugation, the bacterial cells were washed and re-suspended in 30 μl PBS. Viral nucleocapsid protein (N) (TGEV-N) was detected by Western blotting. PBS used in this test served as a native control, bacteria without virus served as a mock and TGEV served as the positive control.

### The impact of *B. subtilis* or surfactin treatments on IPEC-J2 cells

Some studies reported that probiotic bacteria might also indirectly interfere with virus by altering the state of cells, stimulating innate and adaptive immunity. To find out how the *B. subtilis* or surfactin treatments mediate the state of cells, we made a single-treatment assay. Cells were treated with 1.00E + 07 cfu/ml *B. subtilis* or 0.002 mg/ml surfactin for 1.5 h, then the p-EGFR, APN and TLR-6 proteins were detected by Western blotting.

### RNA extraction and qRT-PCR

For quantitative reverse transcription-PCR (qRT-PCR), total RNA from IPEC-J2 cells was extracted using a TRIzol reagent (Life Technologies) and subjected to reverse transcription with Prime Script qRT-PCR Kit (Takara, Dalian, CA). qPCR reactions were performed in ABI 7500 instrument (Applied Biosystems, U.S.A.). Gene expression was calculated with the comparative *C*_t_ method and normalized to the endogeneous levels of GAPDH. Primers sequences used for qRT-PCR are listed in Table 1. The data were analysed using the ABI PRISM 7500 software tool (Applied Biosystems).

**Table 1:** Primer sequences used for qRT-PCR

Gene	Type	Primer pairs (5′-3′)
GAPDH-for	Forward	TCATCATCTCTGCCCCTTCT
GAPDH-rev	Reverse	GTCATGAGTCCCTCCACGAT
TGEV-for	Forward	CAATTCCCGTGGTCGGAAGA
TGEV-rev	Reverse	TTTACGTTGGCCCTTCACCA
TLR-6-for	Forward	CTTTGCCCACCACAACCTCT
TLR-6-rev	Reverse	TTCACATCATCCTCTTCAGCGAC

### Western blotting

For immunodetection of the TGEV-N, p-EGFR, APN and TLR-6 proteins by Western blotting [20], rabbit anti-TGEV (VMRD, Hangzhou, China), rabbit anti-p-EGFR (CST) and rabbit anti-TLR6 (Bioss), followed by HRP–conjugated goat anti-mouse IgG and HRP–conjugated goat anti-rabbit IgG (Sigma) were used. The signal was detected using Super Signal West Pico lit (Thermo Scientific) and subjected to Image Reader LAS-4000 imaging system (FUJIFILM, Japan). The intensity of the bands in terms of density was measured and normalized against GAPDH expression. Three independent experiments and appropriate gel exposures yielded very similar results for each treatment modality.

### Apoptosis assay

At indicated times in the three different treatment assays (the pre-, co-, out-treatment assays), cell apoptosis was further analysed with FITC Annexin V/propidium iodide (PI) staining assay (Miltenyi Biotec, Shanghai, China) as described recently [21].

### Statistical analysis

Results are expressed as means ± S.D. or S.E.M.. One-way ANOVA and Student’s *t* test were employed to determine statistical differences among multiple groups. A *P* value of <0.05 was considered to be significant (*P<0.05, **P<0.01).

## Results

### The safe dose of *B. subtilis* or surfactin

**Figure 1:**
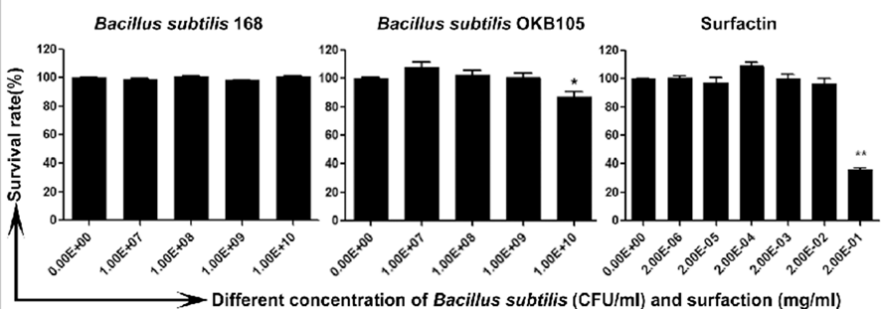
The cytotoxicity of *Bacillus subtilis* or surfactin in IPEC-J2 cells. * B. subtilis* or surfactin were added to confluent cells in a 96-well plate, which were then incubated at 37°C for 2 h. Cell viability was measured by MTT and normalized to the value of non-treated control cells (set at 100%). The cell survival rates at different concentrations are given and 50% above the cell survival rate is regarded as a safe dose. Data are expressed as the S.E.M. from three independent experiments.

It was necessary to ensure that the doses added to cells were non-toxic. As shown in Figure 1, *B. subtilis* 168 was non-toxic in the used doses, *B. subtilis* OKB105 was non-toxic up to 1.00E + 09 cfu/ml, and the safe dose of surfactin was up to 0.02 mg/ml. Therefore, the safe dose of *B. subtilis* (1.00E + 07 cfu/ml) and surfactin (0.002 mg/ml) were used in the next study.

### *B. subtilis* or surfactin inhibit the entry of TGEV

**Figure 2:**
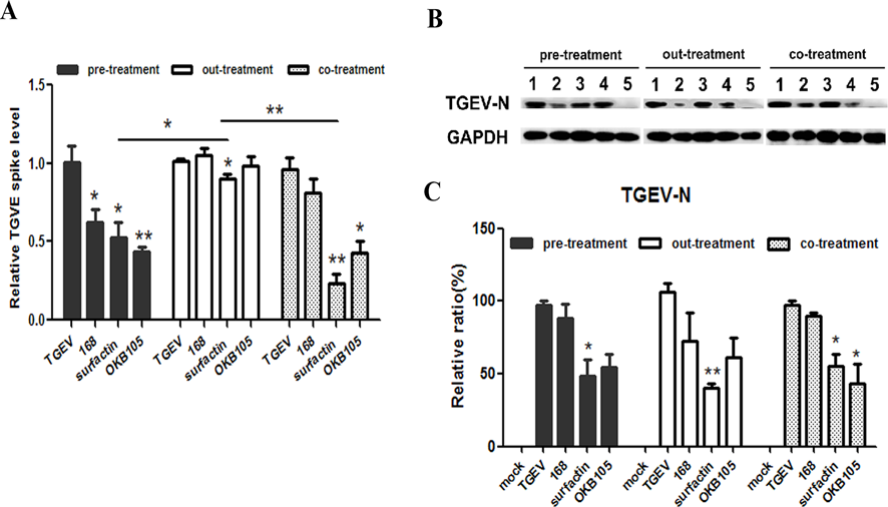
Suppress activity of *B.subtilis* or surfactin. Cells were exposed to *B. subtilis* 168, OKB105 and surfactin in different treatments as described above. For the indicated time points, cells were collected and the yield of virus was determined by qRT-PCR (**A**) and Western blotting (**B**). (B) Lane 1, TGEV control; lane 2, virus from cells treated with 0.002 mg/ml surfactin; lane 3, virus from cells treated with 1.00E + 07 cfu/ml *B. subtilis* 168; lane 4, virus from cells treated with 1.00E + 07 cfu/ml *B. subtilis* OKB105; lane 5, mock. (**C**) Mean relative protein ratio of TGEV-N. Blots were reported with antibody to GAPDH as a loading control. The mean ± S.D. from three independent experiments are shown. Significance levels for the differences between *B. subtilis* and surfactin treatments and virus control from untreated cells are given above the bar: *P<0.05, **P<0.01.

After different treatments, we detected the levels of *TGEV-N* mRNA and protein expression. First, for different ‘drugs’, our results showed that the relative amounts of viral RNAs in the surfactin-treated IPEC-J2 cells decreased in all treatments. On the other side, OKB105 reduced the relative amounts of viral RNAs in the pre-treatment and co-treatment, where cells existed. While *B. subtilis* 168 could only decrease the relative amounts of viral RNAs in the pre-treatment (Figure 2A). Second, for different ‘drugs’ in the same treatment, *B. subtilis* OKB105 showed the best suppression activity in the pre-treatment, where it had enough time of interacting with the cells. However, in the out-treatment, where the cells were not present, *B. subtilis* did not show significant inhibition, while surfactin did. Similar results were obtained in the Western blotting analysis (Figure 2B). Taken together, these data indicated that there might be a hidden association between *B. subtilis* and IPEC-J2 cells, while the surfactin might function on both the virus and the cells, and *B. subtilis* and the surfactin might show synergetic effect to some extent.

### The reduction in the virus infectivity by the surfactin

**Figure 3:**
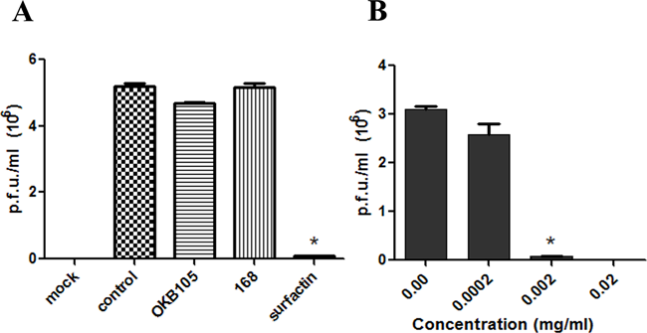
Evaluation of the *B. subtilis* or surfactin antiviral activities using plaque formation assay. (**A**) Virus load expressed as pfu/ml was significantly reduced after treatment with surfactin compared with untreated virus. (**B**) The inhibitory effect of surfactin on TGEV was dose dependent (independent-samples T test, *P*<0.01).

To detect whether surfactin could directly reduce the infectivity of TGEV, a plaque assay was performed. The results showed significant (*P*<0.01) reduction in the TGEV load after treating with 0.002 mg/ml surfactin, but *B. subtilis* 168 or OKB105 did not (Figure 3A). Subsequently, the inhibitory effect of surfactin was further examined by mixing TGEV with different doses of surfactin, and the result showed that the reduction was in a dose-dependent manner (Figure 3B).

### Attachment of TGEV particles to *B. subtilis*

**Figure 4:**
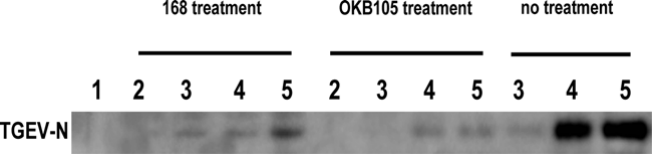
Attachment of TGEV particles to *B. subtilis* 168 and OKB105. After incubation with different concentration of TGEV ( MOI=0.01, 0.1, 0.2) for 1.5h, B.subtilis 168 and OKB105 were washed and detected by the TGEV-N in the western blotting analysis. Lane 1, negative control, the PBS used to re-suspend the bacterial cells; lane 2, mock, that bacterial cells without virus; lane 3 to lane 5, treatment with different concentration of TGEV (MOI=0.01, 0.1, 0.2).. No treatment: positive control, TGEV without bacterial treatment.

A cell-free assay was performed to survey the attachment of TGEV to the *B. subtilis.* As shown in Figure 4, virus particles were bound by *B. subtilis* 168 and OKB105. And when mixed with TGEV at MOI 0.01, *B. subtilis* 168 could attach much more virus than OKB105.

### *B. subtilis* suppresses the TGEV entry by competing with virus for its receptors and improving the state of the IPEC-J2 cells

#### *B. subtilis* competes with TGEV for the viral-entry receptors

**Figure 5:**
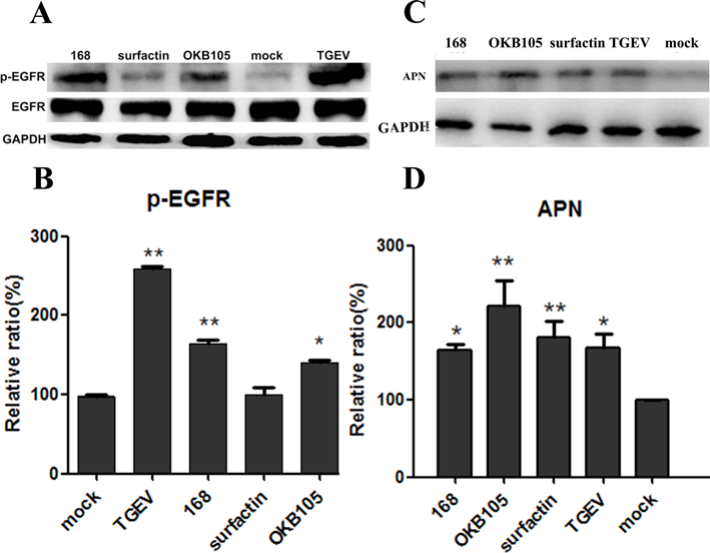
*B. subtilis* 168 and OKB105 enhance the expression of APN protein and p-EGFR in IPEC-J2 cells. IPEC-J2 cells were treated with *B. subtilis* or surfactin respectively, for 1.5 h and cell lysates were analysed for the expression of p-EGFR and APN protein. Both the TGEV and *B. subtilis* 168 and OKB105 enhanced the EGFR activation, and increased the expression of APN. Blots were reported with antibody to GAPDH as a loading control. (**A**) Expression of p-EGFR at IPEC-J2 in the protein level. (**C**) Expression of APN at IPEC-J2 in the protein level. (**B**) Mean relative protein ratio of p-EGFR. (**D**) Mean relative protein ratio of APN.

Binding to the cellular receptor is the first step of CoV entry process [22,23]. To test whether our ‘drugs’ could attach to the viral-entry receptors, we performed a single-treatment experiment. Interestingly, results showed that after stimulation with *B. subtilis*, both 168 and OKB105, the expression of both APN protein (Figure 5C) and p-EGFR (Figure 5A) were increased, which was the similar effect with the TGEV treatment. However, the surfactin stimulus did not change the two receptors expression.

#### *B. subtilis* up regulate the expression of TLR-6 in IPEC-J2 cells

**Figure 6:**
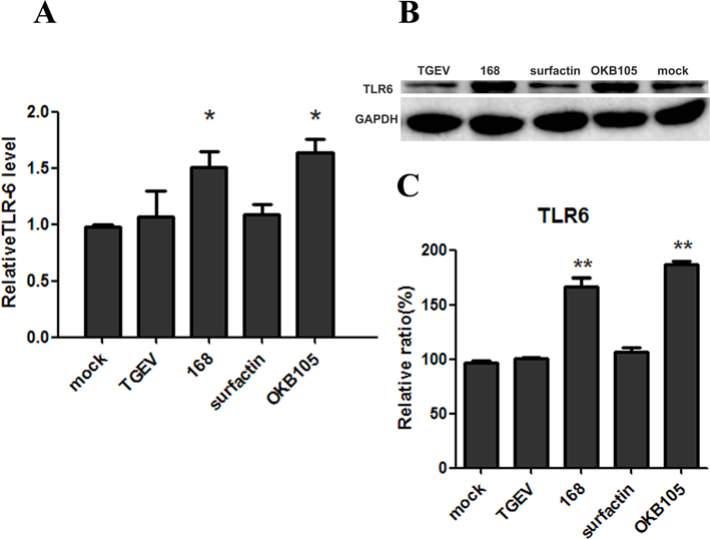
Expression and regulation of TLR-6 at IPEC-J2 cells in the mRNA (A) and protein (B) level. IPEC-J2 cells were left either non-stimulated (mock) or were stimulated with *B. subtilis* or surfactin. (A) RT-PCR was employed to determine *TLR-6* mRNA expression. Data are shown as mean TLR-6/GAPDH ratio. (B) Mean relative protein ratio of TLR-6.

The state of cells is important to resist the pathogens. As the data shown in Figure 6, stimulation with with *B. subtilis* 168 or OKB105 could significantly up-regulate the *TLR-6* mRNA expression in IPEC-J2 cells (Figure 6A). This could be demonstrated by Western blot on the protein level (Figure 6B).

#### *B. subtilis* and the surfactin decreased the percentage of apoptotic cells

**Figure 7:**
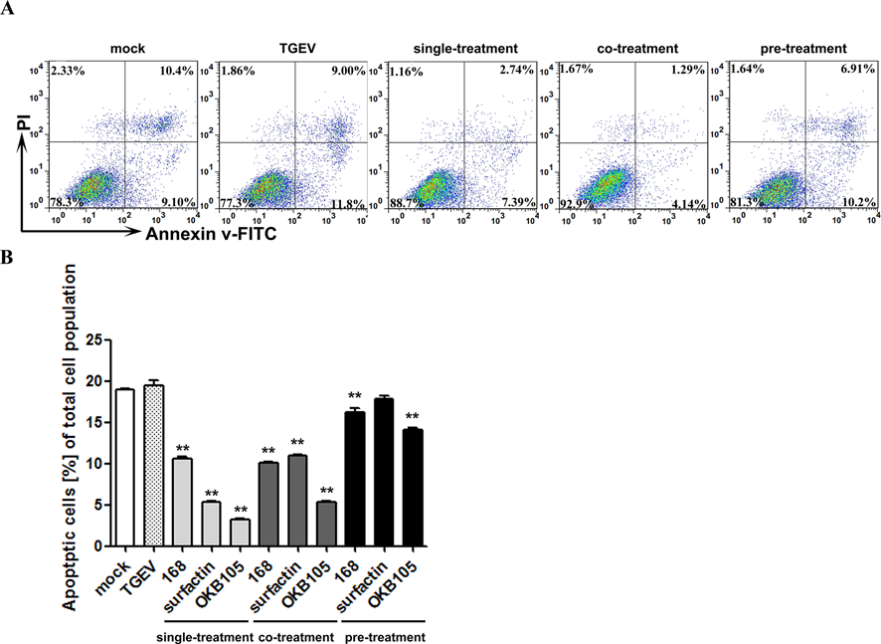
The inhibitory effect of *B. subtilis* or surfactin on IPEC-J2 cells apoptosis. (**A**) Scatter plots of the annexin V-FITC/PI flow cytometry results of a representative experiment are presented below the graphs. The lower right quadrants represent cells in the early stage of apoptosis. The upper right quadrants represent cells in the later stage of apoptosis or necrotic cells. (**B**) The are expressed as the percentage of Annexin V-FITC-positive cells (apoptotic cells) in different treatments (*P<0.05, **P<0.01, compared with the data from mock-infected cells).

To explore the protective effect of *B. subtilis* and surfactin, the apoptosis of IPEC-J2 cells was assessed. As shown in Figure 7, TGEV could increase the apoptosis level of IPEC-J2 cells to some extent, while *B. subtilis* and surfactin could significantly reduce the apoptotic cells number (*P*<0.01). Although the ‘drugs’ displayed the same effect in the co-treatment, but the reduction extent was less than that in the single treatment. We owned this phonmenon to that the apoptosis caused by TGEV, which means when TGEV existed, the apoptosis level of IPEC-J2 was higher, and the reduction extent of apoptpsis our ‘drugs' caused was ease..

## Discussion

TGEV enters epithelial cells by binding to the cellular receptor and then mediates membrane fusion at the plasma membrane or by endosomal uptake [24,25]. Previous studies have proven that APN protein is the receptor of TGEV [26]. Recently, Hu et al. [24] determined that EGFR was another receptor for TGEV entering IPEC-J2 cells. In order to interrupt TGEV infection in the origination stage, we detected the inhibition effect of *B. subtilis* OKB105 and the surfactin on TGEV entry process *in vitro*.

Different experimental protocols were applied in the present study, the pre-, co- and out-treatment assays. The cells were challenged with TGEV at MOI 0.01, as this more closely reflects the natural infection [27]. The results showed that surfactin could reduce the virus yields in all processes, no matter whether the host cells existed or not, while *B. subtilis* OKB105 only had the antiviral activity when the cells existed (Figure 2A) . We conjectured that surfactin could both affect the virus and the cells, while *B. subtilis* might alter the state of cells, eventually leading to an antiviral response. This hypothesis was confirmed in the sequential analysis.

Many reports showed that the probiotics could trap the virus by drop in virus titres [27,28]. Our results were consistent with these observations, after incubating with different titres of TGEV, *B. subtilis* 168 and OKB105 could trap most of TGEV on their surface (Figure 4). And an interesting phenomenon was that the attachment ability of *B. subtilis* 168 was better than *B. subtilis* OKB105, for there was much virus on the *B. subtilis* 168 surface when mixed with TGEV at MOI 0.01, which might indicate that the surfactin secreted by *B. subtilis* OKB105 had destroyed the trapped virion so that could not be detected by the Western blotting. This hypothesis was confirmed in the plaque assay, and we also confirmed that the inactivity of surfactin was dose dependent (Figure 3). Similar results had been observed in previous studies, by using EM, Dirk Vollenbroich et al. detected that the lipid membrane of SHV-1 was disintegrated after incubated with surfactin at 37°C for 1 h, Kracht et al. [12] also reported that surfactin could inactivate VSV.

Evidence have shown that probiotics could block viral attachment by competitive inhibition if they were able to bind viral receptors at the surface of cells [29,30]. Basbaum et al. [31] demonstrated that Gram-positive bacteria could active the EGFR by their lipoteichoic acid. Similarly, in the present study, we found that after stimulating with *B. subtilis* 168 and OKB105 for 1.5 h, the phosphorylation of EGFR and the expression of APN protein were both increased (Figure 6), which indicated that *B. subtilis* might compete with TGEV for binding to the receptors at the surface of IPEC-J2 cells.

The states of cells are critical for keeping healthy, including the response ability and the balance between intestinal cell proliferation and apoptosis, and TLRs play an important role in the sensing the viruses and in the initiation of antiviral host-defence response [32,33]. Since our study was focused on the TGEV entry process, the TLRs at the cells’ surface were investigated. To our knowledge, IPEC-J2 cells can express TLR1, TLR2, TLR3, TLR4, TLR6, TLR8, TLR9 and TLR10, but only TLR1, TLR2, TLR4 and TLR6 were expressed at the cells’ surface [34,35]. Regretfully, the TLR-2 was not detected in our IPEC-J2 cells (results not shown). Surprisingly, our results showed that the expression of TLR-6 was up-regulated after incubation with *B. subtilis* 168 and OKB105 (Figure 6). The results were supported by the fact that TLR-6 was a TLR that could identify the lipoproteins of Gram-positive bacteria [36]. TLR-6 was reported as a novel member of TLRs by Takeuchi et al. [37] in 1999, and it consisted of the signalling pathway of TLR2–TLR6–MyD88, MDA-5–IPS-1 and NALP3 inflammasome pathways [38].

Several clinical studies had demonstrated that TGEV could induce the apoptosis of some kinds of cells like porcine kidney (PK-15) cells [39,40] and ST cells [41]. Additionally, studies have reported that apoptosis was an important regulatory mechanism in intestine maturation [42]. In the present study, after a short-time incubation, all the three treatments could depress the percentage of apoptotic cells (Figure 7). And the depression of the percentage of apoptotic cells was better in the single treatment, where the TGEV did not exist, which might indicate that the TGEV could induce the apoptosis of this IPEC-J2 cells to some extent. Interestingly, Kim et al. [43] determined that surfactin could induce pro-apoptotic of LoVo cells, a human colon carcinoma cell line, when treated for 24 h. While in our study, we found that after treating with 0.002 μg/ml surfactin for 1.5 h, the percentage of apoptotic cells was depressed, which indicted that a safe dose and for a safe time, surfactin could display a positive effect on cells. But how surfactin affect the cells, especially animal cells needs more penetrating study.

## Conclusion

The results of the present study demonstrate that *B. subtilis* OKB105 and the surfactin have antiviral activity against TGEV entering IPEC-J2 cells. And that possibly overlapping mechanisms lead to the antiviral activity: might by competing with TGEV in combining to the receptors, adsorptive trapping, inactivation of virus particles of surfactin, improvement of the cell state through activating the innate immunity and induce the apoptosis level. This finding suggests that *B. subtilis* OKB105 and the surfactin could serve as potential candidates against TGEV entry *in vitro*.

## Abbreviations

APN: aminopeptidase N
cfu: colony forming unit
CoV: coronavirus
CST: Cell Signaling Technology
DMEM: Dulbecco’s modified Eagle’s medium
EGFR: epidermal growth factor receptor
for: forward
GAPDH: glyceraldehyde-3-phosphate dehydrogenase
HRP: Horseradish Peroxidase
IPEC-J2: intestinal porcine epithelial cell line
IPS-1: interferon beta promoter stimulator 1
LB: Luria broth
LoVo: human colon cancer cell line LoVo
MDA-5: melanoma differentiation associated gene 5
MOI: multiplicity of infection
MyD88: myeloid differentiation factor 88
NALP3: NACHT, LRR and domains-containing protein 3
pfu: plaque forming units
PI: propidium iodide
qPCR: quantitative real-time PCR
qRT-PCR: quantitative reverse transcription-PCR
rev: reverse
SHV-1: suid herpes virus type 1
SI: stimulatory index
ST: swine testis
TGE: transmissible gastroenteritis
TGEV: transmissible gastroenteritis virus
TLR: toll-like receptor
T test: student's t test
VMRD: Veterinary Medical Research & Dev
VSV: vesicular stomatitis virus
1640 medium: culture medium for ST cells
